# Quantitative anatomy of the fourth ventricle floor: a cadaveric morphometric study

**DOI:** 10.1007/s11845-025-04170-5

**Published:** 2025-11-24

**Authors:** Ufuk Erginoglu, Umid Sulaimanov, Irem Uslu, Cagdas Ataoglu, Abdullah Keles, Abdurrahman Aycan, Mustafa K. Baskaya

**Affiliations:** Department of Neurological Surgery, University of Wisconsin School of Medicine & Public Health, 600 Highland Avenue, Madison, WI 53792 USA

**Keywords:** Brainstem, Facial Colliculus, Fourth Ventricle, Hypoglossal Trigone, Rhomboid Fossa, Vagal Trigone

## Abstract

**Background:**

The floor of the fourth ventricle contains critical surface landmarks overlying brainstem nuclei. Despite advances in microsurgical techniques, detailed morphometric data on these landmarks remain limited.

**Aims:**

To perform a morphometric analysis of the fourth ventricle floor using cadaveric specimens, with emphasis on dimensions, symmetry, and proportional relationships relevant to surgical planning.

**Methods:**

Forty formalin-fixed adult human brainstems were used. Sixteen anatomical parameters were assessed, including twelve linear distances and four qualitative observations. Key landmarks—including the facial colliculus, sulcus limitans, striae medullares, and the hypoglossal and vagal trigones—were measured under magnification. Anatomical ratios were calculated, and findings were compared to prior cadaveric studies.

**Results:**

The mean floor length and width were 33.58 ± 2.49 mm and 20.0 ± 1.83 mm, respectively. The facial colliculus consistently divided the floor into symmetrical rostral and caudal segments (~ 43% each). The hypoglossal and vagal trigones, measured for the first time, occupied 25.44% and 12.69% of the caudal floor length. The safe midline corridor between sulci limitans comprised only 30.59% of the floor width. Statistically significant asymmetry was found in lateral recess lengths (*p* = 0.003) and striae medullares bundle counts (*p* = 0.044). Most measurements differed significantly from previously published data (*p* < 0.001).

**Conclusion:**

This is the first cadaveric study to define the hypoglossal and vagal trigones quantitatively. The findings clarify the topographic relationships of safe and high-risk surgical corridors in the fourth ventricle floor and provide normative data to support safer microsurgical planning.

## Introduction

The fourth ventricle forms the posterior segment of the ventricular system and lies along the dorsal surface of the brainstem, bordered anteriorly by the pons and medulla and posteriorly by the cerebellum. Its floor, or rhomboid fossa, contains critical surface landmarks overlying cranial nerve nuclei and brainstem pathways. These include the facial colliculus, sulcus limitans, medial eminence, striae medullares, and surface elevations such as the hypoglossal and vagal trigones. Precise knowledge of these anatomical features is essential in neurosurgical procedures involving midline suboccipital or paramedian surgical corridors [1–9].

In light of this anatomical complexity, various surgical corridors have been developed to access fourth ventricular lesions. Among them, telovelar, subtonsillar, and transvermian approaches remain the most widely used. The telovelar and subtonsillar routes utilize natural planes through the cerebellomedullary fissure and beneath the tonsils, preserving midline structures and minimizing cerebellar injury [6, 10, 11]. In contrast, the transvermian approach—historically favored for its direct midline exposure—has declined due to its association with cerebellar mutism and damage to midline structures. More recently, superior transvelar and keyhole techniques have emerged to target rostral or dorsal brainstem lesions through minimally invasive access, though their applicability remains anatomically constrained. Regardless of technique, surgical corridor selection depends on the rostrocaudal level of the lesion, its lateral extension, and relationships to surrounding structures such as the cerebellar peduncles and lateral recesses [1, 3, 6, 8, 12–16].

These approaches are frequently employed to treat diverse pathologies located within or adjacent to the fourth ventricle and brainstem. Typical lesions include ependymomas, subependymomas, medulloblastomas, cavernous malformations, and hemangioblastomas—all of which may distort local anatomy and require careful dissection near regions that contain vital cranial nerve nuclei[13, 14, 16–20]. The median sulcus along the midline floor of the fourth ventricle is often used as a surgical entry point due to its relative distance from critical motor and sensory nuclei, which lie lateral to the sulcus limitans. Various safe entry zones have been proposed in this context, including the suprafacial, infrafacial, and pericollicular triangles [1, 4, 6, 8, 10, 12, 17, 21, 22]. While these strategies are grounded in surface anatomy, they remain limited by a lack of quantitative cadaveric data regarding the true size, symmetry, and spatial relationships of the floor’s key landmarks.

Previous cadaveric studies have reported general morphometry of the fourth ventricle, such as total floor length and transverse width [1–3, 7–9]. However, discrepancies in anatomical definitions—such as variation in the precise aqueductal opening or lateral boundary—have limited their reproducibility. Furthermore, none of these studies have quantitatively characterized the hypoglossal or vagal trigones, despite their role as visible markers of underlying cranial nerve nuclei. Similarly, while sulcus limitans and striae medullares are frequently cited in surgical discussions, their measurable dimensions, symmetry, and variability have not been systematically documented [1, 3, 4].

The aim of this study is to provide a comprehensive morphometric analysis of the floor of the fourth ventricle using formalin-fixed human cadaveric brainstems. We seek to define the dimensions of key anatomical landmarks—including the rostrocaudal length and mediolateral width of the ventricular floor, lateral recesses, sulci, trigones, and surface fibers—and to derive anatomical ratios that reflect their proportional relationships. Although surgical relevance is considered, the primary objective is to establish normative morphometric data that may guide safer planning and interpretation of midline suboccipital approaches to the fourth ventricle. This study offers the first reported cadaveric measurements of the hypoglossal and vagal trigones and provides new insights into the spatial organization of the ventricle floor for neurosurgeons, anatomists, and educators.

## Materials and methods

**Fig. 1:**
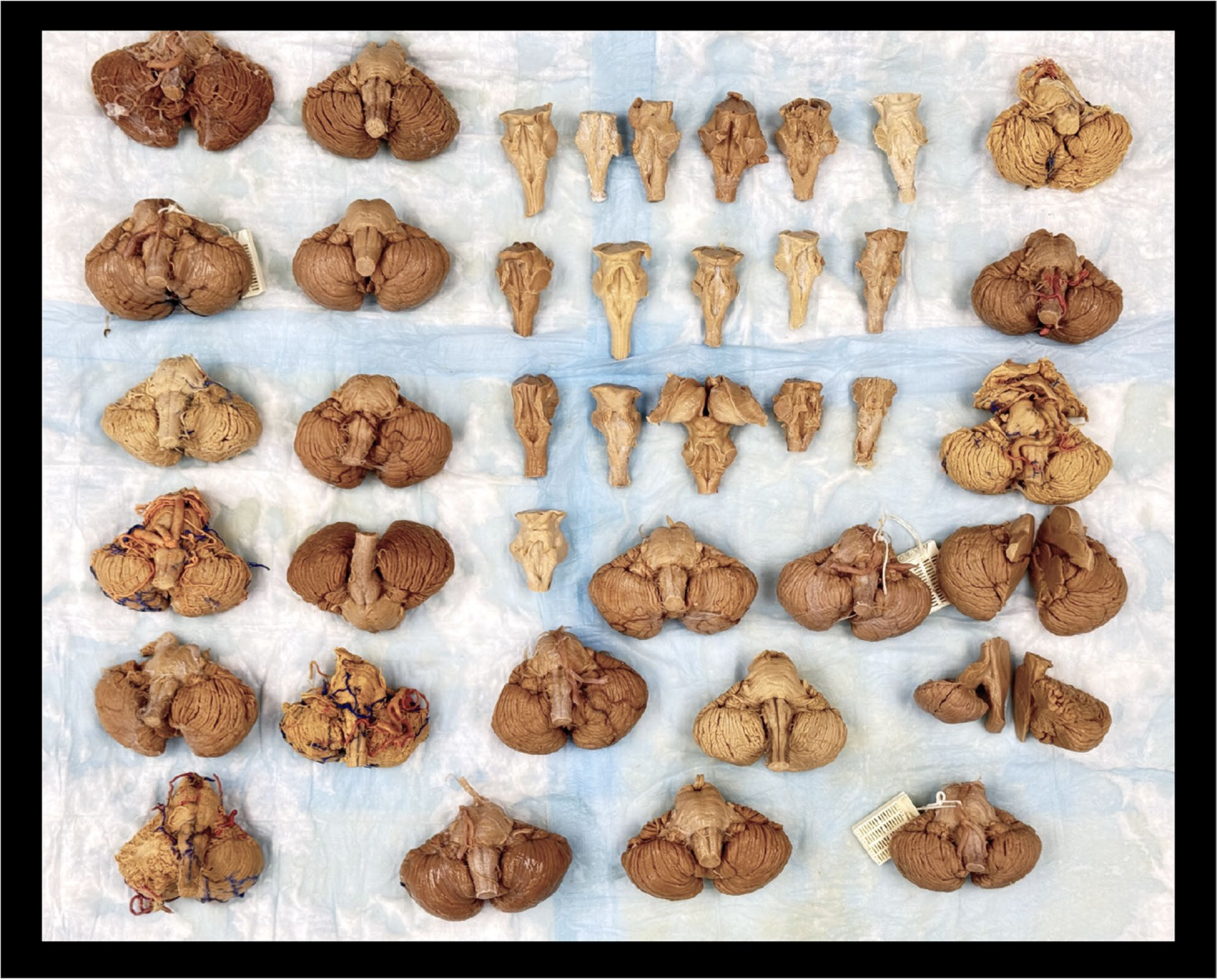
Macroscopic view of all 40 formaldehyde-fixed adult human cadavers. Some show intact posterior fossa contents—including cerebellum, brainstem, and fourth ventricle—prior to exposure, while others display the fully revealed fourth ventricular floor following removal of the cerebellar vermis, overlying lobules, and tela choroidea. This figure illustrates the anatomical preservation and exposure range across the cadaveric cohort used for morphometric analysis

**Fig. 2:**
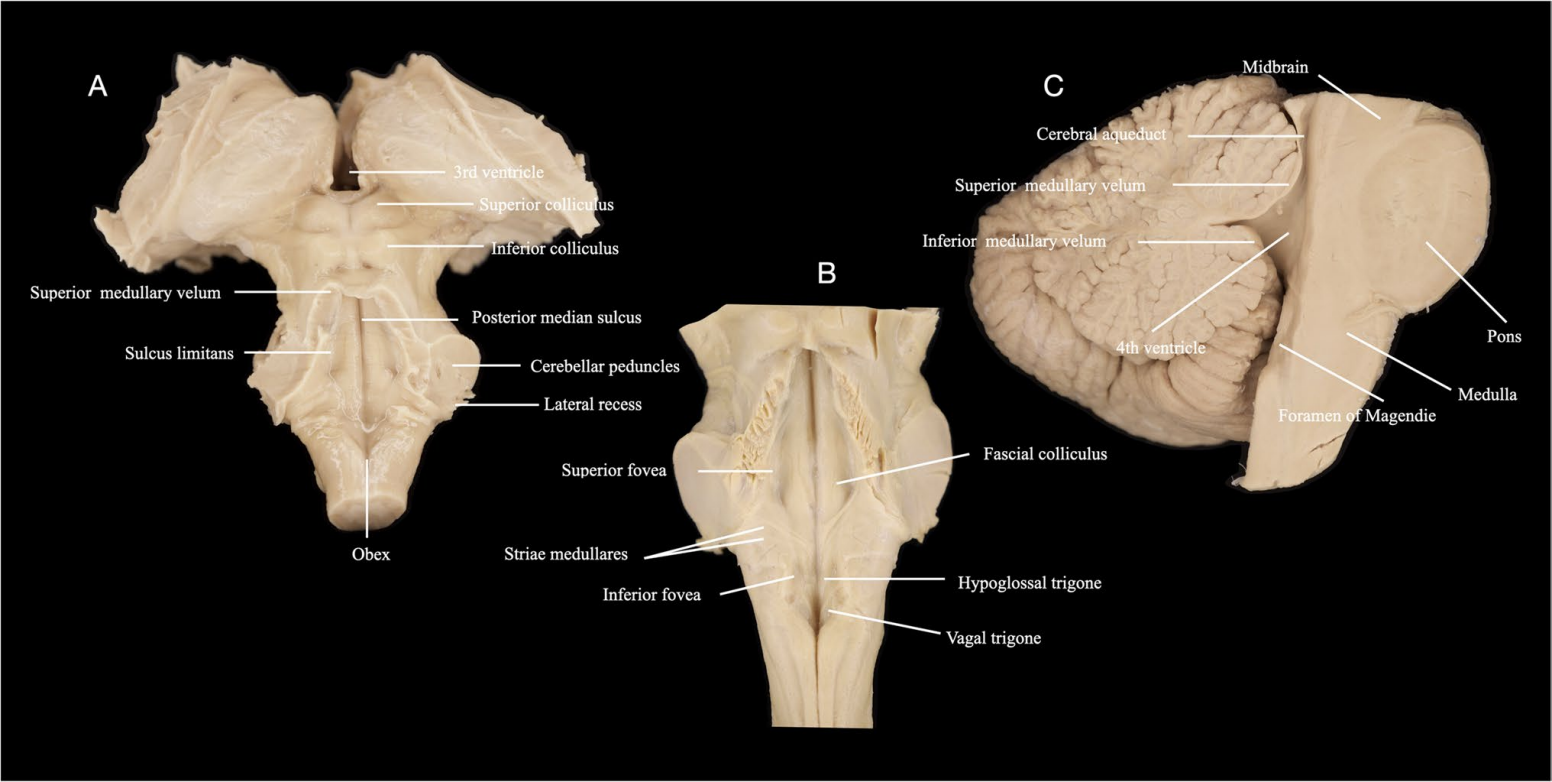
Anatomical views of the fourth ventricle in selected cadaveric specimens. (**A**) Dorsal view showing the superior and inferior colliculi, superior medullary velum, posterior median sulcus, sulcus limitans, cerebellar peduncles, lateral recess, obex, among others. (**B**) Coronal view of the fourth ventricular floor demonstrating bilateral facial colliculi, hypoglossal and vagal trigones, striae medullares, superior and inferior foveae, among others. (**C**) Mid-sagittal section showing the cerebral aqueduct, fourth ventricle, superior and inferior medullary vela, and foramen of Magendie, among others, highlighting the rostrocaudal axis of the ventricular system and its relationship to the cerebellum and tectum

This morphometric study was conducted on 40 formaldehyde-fixed adult human cadavers. Each cadaver contained an intact brainstem, cerebellum, and associated posterior fossa structures, with all cranial bones removed to allow full exposure (Fig. 1). The floor of the fourth ventricle was accessed by carefully removing the cerebellar vermis, overlying lobules, and tela choroidea when necessary. Microdissection was performed under magnification using surgical loupes or a dissecting microscope to ensure precise identification of surface landmarks (Fig. 2). All measurements were obtained under direct visualization using digital calipers.

**Fig. 3:**
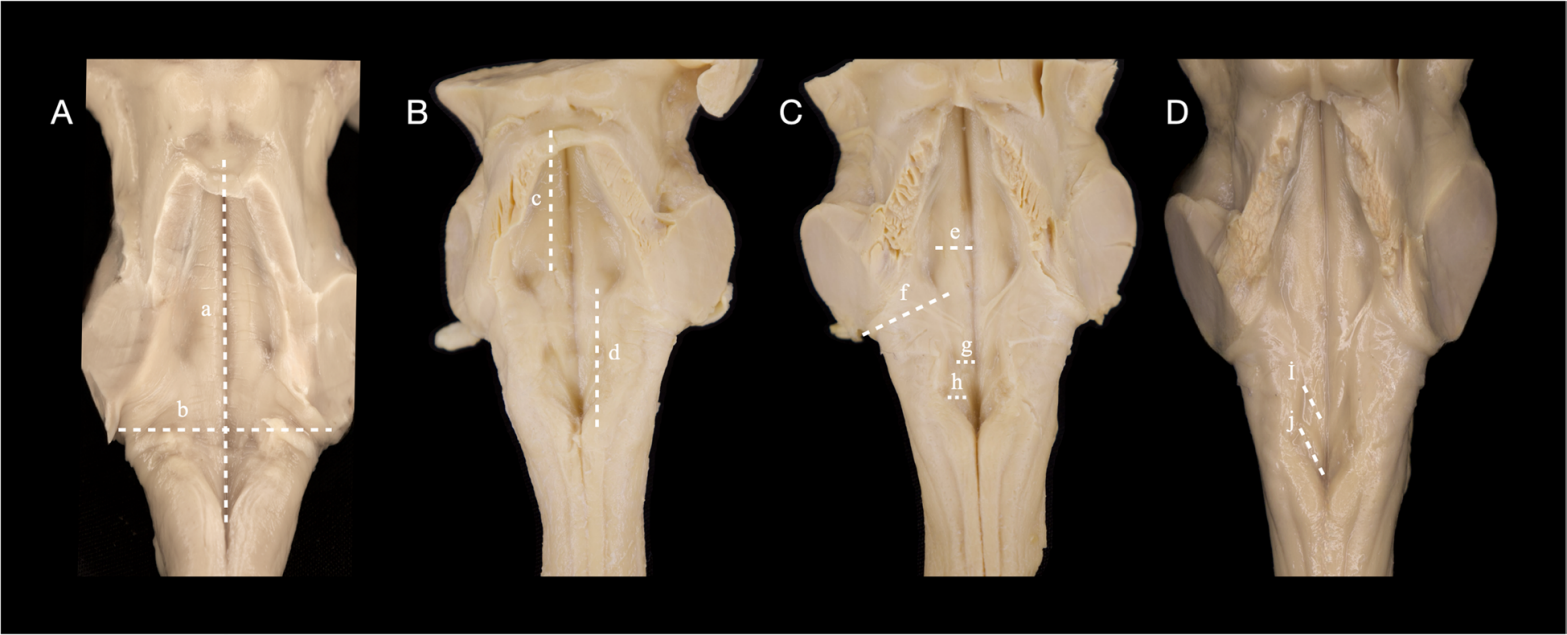
Quantitative morphometric measurements of the fourth ventricular floor. **(A)** **a**: rostrocaudal length from the aqueduct opening to the obex. **b**: transverse width between the foramina of Luschka; **(B)** **c**: distance from the aqueduct to the superior margin of the facial colliculus, representing the rostral (pontine) segment, d: distance from the inferior margin of the facial colliculus to the obex, defining the caudal (medullary) segment; **(C)** **e**: distance from the median sulcus to the sulcus limitans, **f**: lateral recess length measured obliquely from the vestibular area, adjacent to the sulcus limitans, to the foramen of Luschka, **g**: hypoglossal trigone width (transverse axis), **h**: vagal trigone width (transverse axis); **(D)** (**i**) hypoglossal trigone length (rostrocaudal axis), (**j**) vagal trigone length (rostrocaudal axis)

Sixteen anatomical parameters were assessed. Twelve were linear distances recorded in millimeters, while four were qualitative or ordinal observations. All measurements were performed in situ, with the brainstem fixed in a neutral position to avoid distortion of surface geometry. The rostrocaudal length of the fourth ventricle floor was measured from the aqueduct opening at the level of the superior medullary velum to the obex. The transverse width was recorded between the right and left foramina of Luschka, marking the lateral extent of the lateral recesses. To divide the ventricular floor into functional zones, the distance from the aqueduct to the superior margin of the facial colliculus was measured to represent the rostral (pontine) floor, and the distance from the inferior margin of the colliculus to the obex defined the caudal (medullary) segment (Fig. 3).

The lateral recess lengths were measured bilaterally. Each was recorded obliquely from the vestibular area, adjacent to the sulcus limitans, to the foramen of Luschka. Mediolateral dimensions were also recorded. The distance from the median sulcus to the sulcus limitans was measured separately on the right and left sides, at the point where the sulcus limitans was most visible, typically in the mid-pons, where it demarcates the transition from motor to sensory nuclei. The hypoglossal and vagal trigones were evaluated by measuring their maximum width (transverse axis) and length (rostrocaudal axis) on the side where the structure was most clearly distinguishable. These surface elevations correspond to the underlying cranial nerve XII and X nuclei, respectively (Fig. 3).

The presence or absence of the facial colliculi was recorded bilaterally. The number of striae medullares bundles was counted visually on each side using an ordinal 0–4 scale, based on the number of discrete fiber bands identifiable without magnification beyond × 10. No thickness or length measurement was attempted for the striae; only count data were collected. For all paired measurements, both right and left values were recorded independently. In analyses involving structures that could be asymmetric, only the most clearly defined side was included to avoid under- or over-representation (Fig. 2).

Descriptive statistics were calculated for all linear variables, including mean, standard deviation (SD), minimum, and maximum values. The Shapiro–Wilk test was used to evaluate the normality of distribution. Normally distributed paired data (e.g., right vs. left lateral recess length or sulcus limitans width) were compared using the paired t-test. If normality was not met, the Wilcoxon signed-rank test was applied. Comparisons of ordinal data, such as striae count, were also analyzed using the Wilcoxon test. A significance level of *p* < 0.05 was used for all comparisons (Table 1).

**Table 1:** Descriptive morphometric measurements of the fourth ventricle floor in cadaveric specimens (*n* = 40)

Measurement	Right Side (Mean ± SD)	Right Side (Min–Max)	Left Side (Mean ± SD)	Left Side (Min–Max)	p-value
Median Sulcus → Sulcus Limitans	3.09 ± 0.76	2.0–4.5	3.03 ± 0.67	1.5–4.0	0.49
Striae Medullares Count	2.07 ± 1.13	0–4	1.68 ± 1.01	0–4	0.044
Lateral Recess Length	6.64 ± 1.15	5.0–8.0	6.34 ± 1.03	5.0–8.0	0.003
	**Mean ± SD**		**Min–Max**		**-**
Fourth Ventricle Length	33.58 ± 2.49		29.0–39.0		-
Fourth Ventricle Width	20.0 ± 1.83		17.0–24.0		-
Facial Colliculus (Caudal Margin) to Obex	14.5 ± 1.68		12.0–18.0		-
Aqueduct Opening to Facial Colliculus (Rostral Margin)	14.6 ± 1.97		11.0–18.0		-
Hypoglossal Trigone Width	1.92 ± 0.39		1.5–3.0		-
Hypoglossal Trigone Length	3.65 ± 1.06		2.0–6.0		-
Vagal Trigone Length	1.83 ± 0.31		1.0–2.3		-
Vagal Trigone Width	1.56 ± 0.39		1.0–2.0		-

This table summarizes morphometric measurements of the fourth ventricle floor based on 40 cadaveric specimens

Linear distances are presented as mean ± standard deviation (SD) and range (min–max) in millimeters

Bilateral structures were compared using a paired t-test or Wilcoxon signed-rank test, depending on normality

P-values < 0.05 were considered statistically significant

Anatomical ratios were then calculated to evaluate the proportional relationships between substructures of the fourth ventricle floor and to derive clinically relevant geometric indices. These ratios included the trigone lengths and widths as a percentage of the corresponding caudal floor segment or ventricle width, the division of the floor into rostral and caudal segments relative to the total floor length, and the ratio of the usable surgical corridor (defined by the sulci limitans) to the entire floor width. All ratios were expressed as percentages and analyzed for normality using the Shapiro–Wilk test. Mean, SD, range, and 95% confidence intervals were reported (Tables 1 and 2).

**Table 2:** Anatomical ratios of fourth ventricle substructures and their clinical relevance

Ratio	Mean ± SD (%)	Range (%)	95% CI (%)	Shapiro–Wilk p-value	Normal Distribution	Clinical Interpretation
Hypoglossal Trigone Length/Facial Colliculus to Obex Length	25.44 ± 7.61	11.76–40.0	23.08–27.79	0.43	Yes	Extent of CN XII zone in the caudal floor (medial motor risk)
Vagal Trigone Length/Facial Colliculus to Obex Length	12.69 ± 2.44	5.88–16.67	11.93–13.44	0.08	Yes	Extent of CN X zone in the caudal floor (lateral parasympathetic risk)
Hypoglossal Trigone Width/Fourth Ventricle Width (Luschka–Luschka)	9.66 ± 2.1	6.25–15.0	9.01–10.32	0.14	Yes	Proportion of medial floor occupied by CN XII nucleus
Vagal Trigone Width/Fourth Ventricle Width	7.91 ± 1.98	4.35–11.11	7.29–8.52	0.1	Yes	Proportion of lateral floor occupied by CN X nucleus
Facial Colliculus to Obex Length/Fourth Ventricle Floor Length (Aqueduct to Obex)	43.34 ± 5.17	30.77–55.17	41.73–44.94	0.75	Yes	Proportion of the floor relevant to caudal (medullary) surgical access
Aqueduct to Facial Colliculus/Fourth Ventricle Floor Length	43.57 ± 5.57	31.43–56.67	41.79–45.36	0.77	Yes	Proportion of the floor relevant to rostral (pontine) surgical access
Fourth Ventricle Width/Fourth Ventricle Floor Length	59.71 ± 5.34	48.72–75.86	58.06–61.37	0.14	Yes	Overall shape index of floor (transverse vs. longitudinal)
Sulcus Limitans-to-Limitans Width/Fourth Ventricle Width	30.59 ± 6.13	19.44–42.11	28.63–32.55	0.24	Yes	Proportion of floor width between sulci limitans usable for midline access

This table presents key morphometric ratios derived from cadaveric measurements of the fourth ventricle floor (*n* = 40)

Each ratio expresses the proportional relationship between specific substructures (e.g., trigone size, floor width) and major anatomical axes such as the facial colliculus–obex segment or total ventricular floor length. Values are reported as mean ± SD (%), range, and 95% confidence interval (CI). Shapiro–Wilk test p-values were used to assess normality; all ratios followed a normal distribution. The final column summarizes each ratio’s clinical relevance to surgical corridor planning and risk localization

*Abbreviations:* SD, standard deviation; CI, confidence interval

To place our findings in anatomical and historical context, measurements were compared to previously published cadaveric studies, including those by Antar et al. [3], Umarani et al. [2], Lang et al. [1], and Last and Tompsett [9] (Table 3). In these comparisons, bilateral values in our data were summed when necessary to match single composite values reported in the literature. When previous authors provided means and standard deviations, two-sample t-tests were conducted to assess statistical differences from our findings. In older studies without statistical parameters, comparison was descriptive only.

**Table 3:** Comparison of fourth ventricle floor measurements with published cadaveric studies

Measurement (mm)	This Study (Mean ± SD)	Antar et al. [3]	Umarani et al. [2]	Lang et al. [1]	Last &Tompsett [9]	p-value (vs. Antar)	p-value (vs. Umarani)
Fourth Ventricle Length	33.58 ± 2.49	39.84 ± 4.03	M: 41.51 ± 4.88 F: 40.38 ± 2.08	31.7 (28–38)	29 (21–37)	< 0.0001	< 0.0001
Fourth Ventricle Width	20.0 ± 1.83	22.51 ± 2.43	M: 23.39 ± 2.67 F: 24.72 ± 2.05	24.0 (21–26)	17.0 (12–23)	< 0.0001	< 0.0001
Facial Colliculus → Obex	14.5 ± 1.68	20.74 ± 2.13	M: 24.74 ± 2.31 F: 22.44 ± 1.86	–	–	< 0.0001	< 0.0001
Aqueduct → Facial Colliculus	14.6 ± 1.97	20.54 ± 3.35	M: 23.31 ± 3.65 F: 24.10 ± 2.88	–	–	< 0.0001	< 0.0001
Lateral Recess Length (Right)	6.64 ± 1.15	–	–	–	–		
Lateral Recess Length (Left)	6.34 ± 1.03	–	–	–	–	0.0049	0.0343
Lateral Recess Length (R + L)	12.98 ± 1.71	14.83 ± 2.83	M: 17.39 ± 3.06 F: 16.92 ± 2.21	–	–	< 0.0001	< 0.0001
Median Sulcus → Sulcus Limitans (Right)	3.09 ± 0.76	–	–	–	–		
Median Sulcus → Sulcus Limitans (Left)	3.03 ± 0.67	–	–	–	–		
Median Sulcus → Sulcus Limitans (R + L)	6.12 ± 1.33	5.51 ± 0.88	M: 6.57 ± 0.98 F: 6.42 ± 0.72	–	–	0.0049	0.0343

This table compares morphometric measurements from the present study (*n* = 40 cadavers) with previously reported cadaveric data from Antar et al. (2019), Umarani et al., Lang et al. (1991), and Last & Tompsett (1953)

Measurements are presented as **mean ± SD** or **range**. **Two-sample t-tests** were used to compare our values with reported means; all comparisons were statistically significant (***p* < 0.001**). Differences may reflect variability in the definitions of anatomical landmarks, measurement techniques, and sample populations across studies

*Abbreviations:* SD, standard deviation

## Results

The mean length of the fourth ventricle floor from the aqueduct opening to the obex was 33.58 ± 2.49 mm (range: 29.0–39.0 mm), and the transverse width from the right to the left foramen of Luschka was 20.0 ± 1.83 mm (range: 17.0–24.0 mm). These two principal axes define the overall shape of the floor, with the longitudinal axis establishing the surgical depth and the transverse axis defining exposure width (Table 1).

Rostrocaudal segmentation of the floor revealed nearly symmetrical division around the facial colliculus. The distance from the aqueduct to the facial colliculus, representing the rostral floor segment, measured 14.6 ± 1.97 mm, while the facial colliculus to obex distance, corresponding to the caudal segment, measured 14.5 ± 1.68 mm. This equal division validates the facial colliculus as a consistent surgical landmark in defining pontine versus medullary approaches (Table 1).

The width from the median sulcus to the sulcus limitans measured 3.09 ± 0.76 mm on the right and 3.03 ± 0.67 mm on the left, with no statistically significant difference (*p* = 0.49). These mediolateral distances represent the usable midline floor between sensory and motor columns and were highly symmetrical across sides (Table 1). In contrast, the lateral recess lengths showed mild asymmetry: 6.64 ± 1.15 mm on the right versus 6.34 ± 1.03 mm on the left, a statistically significant difference (*p* = 0.003). Although small in magnitude, this asymmetry may influence lateral corridor exposure in telovelar approaches (Table 1).

The facial colliculus was present bilaterally in all 40 cadavers. The striae medullares were consistently present but varied in number. The right side displayed a mean of 2.07 ± 1.13 bundles, while the left had 1.68 ± 1.01. This difference was statistically significant (*p* = 0.044), suggesting asymmetry in surface fiber organization. However, correlation analysis between total striae count and fourth ventricle width yielded no significant relationship (ρ = 0.03, *p* = 0.85), indicating that bundle density does not scale with cavity size (Table 1).

The hypoglossal trigone had a mean width of 1.92 ± 0.39 mm and a length of 3.65 ± 1.06 mm. The vagal trigone was smaller, measuring 1.56 ± 0.39 mm in width and 1.83 ± 0.31 mm in length. To our knowledge, these are the first quantitative measurements of the hypoglossal and vagal trigones based on cadaveric dissection. While prior literature has described these subependymal structures anatomically, no previous study has reported their precise morphometric dimensions in terms of width and length. These values define the anatomical extent of the cranial nerve XII and X zones within the floor of the fourth ventricle (Table 1).

Several anatomical ratios were derived from these measurements to assess spatial relationships. The hypoglossal trigone occupied 25.44 ± 7.61% of the caudal floor (i.e., the facial colliculus to obex segment), while the vagal trigone accounted for 12.69 ± 2.44%. These ratios quantify the rostrocaudal proportions of the motor and parasympathetic danger zones within the medullary floor. Transversely, the hypoglossal and vagal trigones occupied 9.66% and 7.91% of the total ventricular width, respectively, further defining medial and lateral corridor risks. The floor width between sulcus limitans landmarks comprised 30.59 ± 6.13% of the total ventricle width, representing the midline corridor accessible for surgical entry. The ratio of ventricle width to floor length was 59.71 ± 5.34%, indicating a flat floor with a broad working axis. The facial colliculus was positioned near the mid-floor point, with the rostral (aqueduct to colliculus) and caudal (colliculus to obex) floor zones each comprising approximately 43% of the total floor length. All ratios followed normal distribution (Table 2).

Statistical comparisons with previously published cadaveric data demonstrated significant differences across nearly all comparable measurements (*p* < 0.001; Table 3).

## Discussion

### Overview of morphometric objectives

This cadaveric study aimed to generate normative morphometric data for the floor of the fourth ventricle, with a direct emphasis on anatomical definition and surgical relevance. While surface structures such as the facial colliculus, sulcus limitans, and striae medullares are routinely described in classical neuroanatomy, few prior investigations have quantitatively characterized their spatial organization or proportions [1–3]. Even fewer studies have defined these features in a way that facilitates neurosurgical planning. Our findings clarify the geometric organization of the rhomboid fossa and provide quantitative context for regions commonly referenced in fourth ventricular and dorsal brainstem surgery [6, 7, 10, 15].

### Floor dimensions and surgical segmentation

The rostrocaudal length of the fourth ventricular floor, from the aqueduct opening to the obex, measured 33.58 ± 2.49 mm, while the transverse width, defined by the distance between the foramina of Luschka, was 20.0 ± 1.83 mm. These primary axes delineate the working depth and lateral extent of the surgical field [1–3, 7, 15]. Internally, the floor was nearly symmetrically divided by the facial colliculus into rostral (14.6 ± 1.97 mm) and caudal (14.5 ± 1.68 mm) halves, validating its use as a midpoint reference for distinguishing pontine from medullary zones [6, 8, 11, 15, 21] (Table 1).

Clinically, this symmetric segmentation reinforces the facial colliculus as a structural “equator” of the floor, guiding the use of suprafacial versus infrafacial or pericollicular entry zones [5, 6, 8, 10, 11, 21, 25]. The facial colliculus lies at the junction of motor and sensory transition zones and provides a landmark for orientation during fourth ventricular entry.

### Mediolateral corridor and surface fiber patterns

The distance from the median sulcus to the sulcus limitans averaged 3.09 mm (right) and 3.03 mm (left), with no significant asymmetry (*p* = 0.49) (Table 1). These measurements define a narrow midline corridor that lies medial to both motor and sensory nuclei and is considered relatively safe for dorsal entry [1–4]. Although the region between the median sulcus and sulcus limitans has often been referenced as a potential entry zone, our data support that the true midline—centered on the median sulcus—is preferred to minimize risk to underlying cranial nerve nuclei [6, 8, 10, 15].

The striae medullares were bilaterally visible in all specimens, with significantly more bundles observed on the right (2.07 vs. 1.68, *p* = 0.044) (Table 1). Although functionally benign, dense or asymmetric striae may obscure key anatomical triangles, particularly in the lower pontine region [1–4]. Notably, there was no significant correlation between total striae count and fourth ventricle width (Spearman’s ρ = 0.03, *p* = 0.85), suggesting that bundle density does not scale with overall cavity size.

### Trigone dimensions and high-risk zones

This is the first cadaveric study to report precise dimensions of the hypoglossal and vagal trigones. The hypoglossal trigone measured 1.92 ± 0.39 mm in width and 3.65 ± 1.06 mm in length, while the vagal trigone measured 1.56 ± 0.39 mm by 1.83 ± 0.31 mm (Table 1 and 2). These surface elevations overlie the nuclei of cranial nerves XII and X and define subependymal surgical caution zones during medullary procedures [1–3].

Collectively, these trigones occupied approximately 40% of the caudal floor, severely limiting the usable entry area in this region (Table 2). Only the most well-defined side was measured to avoid under- or overestimation, consistent with prior anatomic practice [1, 3, 4, 6, 8]. Bilateral measurement was deemed unnecessary given their midline or paramedian location and the predominantly unilateral nature of surgical entry.

### Proportional floor ratios and surgical corridor limits

The hypoglossal and vagal trigones occupied 25.44% and 12.69% of the caudal floor length, and 9.66% and 7.91% of the total ventricular width, respectively. In contrast, the usable midline corridor—defined between the sulci limitans—comprised 30.59 ± 6.13% of total floor width (Table 2). These ratios clarify the functional footprint of both surgical caution zones and accessible entry areas [1, 6, 21].

The width-to-length ratio of 59.71% defines the fourth ventricular floor as relatively flat, facilitating microsurgical exposure. The facial colliculus was positioned near the midpoint, with the rostral and caudal segments each comprising approximately 43% of total floor length, reinforcing its landmark utility in planning suprafacial, infrafacial, or pericollicular entry [1, 6, 21].

### Lateral recesses and asymmetry

The lateral recesses showed mild but statistically significant asymmetry: 6.64 mm on the right versus 6.34 mm on the left (*p* = 0.003) (Table 1). This finding may influence side selection for telovelar or subtonsillar approaches, particularly when addressing eccentrically positioned lesions or maximizing lateral exposure. Subtle asymmetry in cerebellomedullary fissure anatomy or foramen of Luschka morphology may underlie these differences [4, 11, 21, 23].

### Comparison with prior morphometric studies

Statistical comparisons with previously published cadaveric data revealed significant differences across nearly all comparable measurements. Our fourth ventricle floor length was shorter than that reported by Antar et al. (39.84 mm) and Umarani et al. (41.51 mm in males), likely due to differing definitions of the aqueduct’s superior boundary [2, 3]. Similarly, our ventricle width was smaller than Lang’s et al. (24.0 mm) but greater than that of Last and Tompsett et al. (17.0 mm), reflecting differing measurement landmarks—namely, the lateral recess versus inferior cerebellar peduncle span [1, 9] (Table 1,2,3). The lateral recess and sulcus measurements in our study were presented bilaterally and combined only for cross-study comparisons, whereas prior works did not clarify laterality. All numerical comparisons against Antar and Umarani datasets showed statistically significant differences (*p* < 0.001), likely due to methodological variation rather than anatomical divergence.

Previous cadaveric studies have documented general dimensions of the fourth ventricle, such as total floor length and transverse cavity width [1–3]. However, these studies vary significantly in their landmark definitions, particularly regarding the location of the aqueduct opening and the lateralmost measurement point for ventricle width. Furthermore, none of these studies have quantitatively characterized the hypoglossal or vagal trigones, despite their importance as surface indicators of cranial nerve nuclei. Similarly, while structures such as the sulcus limitans and striae medullares are routinely referenced in surgical entry discussions, their measurable width, symmetry, and variability remain poorly defined [1, 4].

### Surgical applications and corridor selection

Familiarity with normative fourth ventricle anatomy is essential for safe resection of pathologies such as ependymomas, subependymomas, medulloblastomas, hemangioblastomas, cavernous malformations, and intraventricular lymphomas [10–13, 17–20, 22, 24]. Despite technological advances in neuronavigation and diffusion tensor imaging, precise surface anatomy remains fundamental.

The telovelar approach has become the most widely preferred surgical corridor. It utilizes the natural cerebellomedullary fissure to access the fourth ventricle without disrupting the inferior vermis, thereby preserving midline cerebellar structures and minimizing complications such as ataxia and mutism [5, 6, 11, 12]. The subtonsillar variant expands lateral exposure, while the transvermian approach—historically widely used—is now generally avoided due to its association with cerebellar mutism, disequilibrium, and injury to the fastigial and dentate nuclei. However, in select cases with large tumors extending rostrally toward the aqueduct or compressing the fastigium, it may still be considered when telovelar access is insufficient [11, 12, 22].

More recently, keyhole and superior transvelar approaches have gained favor for targeting dorsal brainstem lesions, though their applicability is anatomically constrained and requires high surgical expertise [12, 16].

### Clinical relevance and limitations

This study establishes foundational morphometric data for the fourth ventricle floor, emphasizing anatomical clarity and surgical planning. By defining dimensions and ratios of common landmarks, our findings can enhance intraoperative decision-making and reduce the risk of cranial nerve injury.

Limitations include the use of formalin-fixed cadavers, which may contract and differ from in vivo conditions. Pediatric and pathological variants were not included. Future studies may incorporate 3D modeling, high-field imaging, and intraoperative correlation to expand clinical applicability.

## Conclusion

The floor of the fourth ventricle demonstrates a consistent geometric organization that can be quantitatively mapped through cadaveric morphometry. Our results define the dimensions and spatial relationships of key subependymal structures, offering a reproducible anatomical framework to support neurosurgical planning, anatomical education, and safer fourth ventricular interventions.

## Data Availability

All data generated or analyzed during this cadaveric study are included in this published article and are available from the corresponding author upon reasonable request.

## References

[1] Lang J Jr., Ohmachi N, Lang J Sr. (1991) Anatomical landmarks of the rhomboid fossa (floor of the 4th ventricle), its length and its width. Acta Neurochir (Wien) 113:84–90. 10.1007/bf01402120 10.1007/BF01402120 1799148

[2] Umarani S, Arjun R, Kumar S, Sivaraj R (2022) Morphometric assessment of the external anatomy of fourth ventricle & dorsal brainstem in South Indian population. Int J Health Sci 6:9609–9614

[3] Antar V, Turk O, Katar S et al (2019) Morphometric assesment of the external anatomy of fourth ventricle and dorsal brainstem in fresh cadavers. Turk Neurosurg 29:445–450. 10.5137/1019-5149.Jtn.24942-18.1 30649830 10.5137/1019-5149.JTN.24942-18.1

[4] Mercier P, Bernard F, Delion M (2021) Microsurgical anatomy of the fourth ventricle. Neurochirurgie 67:14–22. 10.1016/j.neuchi.2018.04.010 29875069 10.1016/j.neuchi.2018.04.010

[5] Mussi AC, Rhoton AL Jr. (2000) Telovelar approach to the fourth ventricle: microsurgical anatomy. J Neurosurg 92:812–823. 10.3171/jns.2000.92.5.0812 10.3171/jns.2000.92.5.0812 10794296

[6] Rhoton AL Jr (2000) Cerebellum and fourth ventricle. Neurosurgery 47:S7-27. 10.1097/00006123-200009001-00007 10983303 10.1097/00006123-200009001-00007

[7] Roesch ZK, Tadi P (2025) Neuroanatomy, Fourth Ventricle. In: StatPearls. StatPearls Publishing 31536186

[8] Copyright © 2025, StatPearls Publishing LLC., Treasure Island (FL),

[9] Zimelewicz Oberman D, Baldoncini M, Campero A (2023) Surgical Anatomy of the Fourth Ventricle. In. pp 391–402. 10.1007/978-3-031-14820-0_20

[10] Last RJ, Tompsett DH (1953) Casts of the cerebral ventricles. Br J Surg 40:525–543. 10.1002/bjs.18004016403 13059333 10.1002/bjs.18004016403

[11] Mussi AC, Matushita H, Andrade FG, Rhoton AL (2015) Surgical approaches to IV ventricle–anatomical study. Childs Nerv Syst 31:1807–1814. 10.1007/s00381-015-2809-0 26351232 10.1007/s00381-015-2809-0

[12] Tanriover N, Ulm AJ, Rhoton AL Jr., Yasuda A (2004) Comparison of the transvermian and telovelar approaches to the fourth ventricle. J Neurosurg 101:484–498. 10.3171/jns.2004.101.3.0484 10.3171/jns.2004.101.3.0484 15352607

[13] Dang DD, Rechberger JS, Leonel L et al (2024) Anatomical step-by-step dissection of midline suboccipital approaches to the fourth ventricle for trainees: surgical anatomy of the telovelar, transvermian, and superior transvelar routes, surgical principles, and illustrative cases. J Neurol Surg B Skull Base 85:172–188. 10.1055/a-2018-4745 38449580 10.1055/a-2018-4745 PMC10914463

[14] Haider AS, McCutcheon IE, Ene CI et al (2023) Subependymomas of the fourth ventricle: to operate or not to operate? J Clin Neurosci 118:147–152. 10.1016/j.jocn.2023.10.025 37944358 10.1016/j.jocn.2023.10.025

[15] Lee HJ, Lee SU, Park E, Kim JS (2024) Cavernous malformation in the fourth ventricle: trivial findings but grave prognosis. Acta Neurol Belg 124:1085–1087. 10.1007/s13760-023-02464-y 38117457 10.1007/s13760-023-02464-y

[16] Spierer R (2023) The debated neuroanatomy of the fourth ventricle. J Anat 243:555–563. 10.1111/joa.13885 37170923 10.1111/joa.13885 PMC10485575

[17] Wang J, Wang X, Xu J et al (2023) Microsurgical management of fourth ventricle lesions via the median suboccipital keyhole telovelar approach. J Craniofac Surg 34:607–610. 10.1097/scs.0000000000008883 35968951 10.1097/SCS.0000000000008883 PMC9944752

[18] Chen T, Ren Y, Wang C et al (2020) Risk factors for hydrocephalus following fourth ventricle tumor surgery: a retrospective analysis of 121 patients. PLoS One 15:e0241853. 10.1371/journal.pone.0241853 33201889 10.1371/journal.pone.0241853 PMC7671531

[19] Hama Y, Sasaki T, Yamoto T et al (2023) Hemangioblastoma of the medulla oblongata that caused isolated fourth ventricle after stereotactic radiosurgery: a case report. Mol Clin Oncol 18:37. 10.3892/mco.2023.2633 37020505 10.3892/mco.2023.2633 PMC10067790

[20] Sufianov RA, Gaysin IA, Iakimov IA, Sufianov AA (2024) Exoscopic removal of the fourth ventricle choroid plexus papilloma with use of a midline suboccipital osteoplastic craniotomy. Neurosurgical Focus: Video 10:V14. 10.3171/2023.10.Focvid23106 38283819 10.3171/2023.10.FOCVID23106 PMC10821634

[21] Zhao J, Zou C, Guo Z et al (2023) Primary central nervous system diffuse large B-cell lymphoma in fourth ventricle: case report and literature review. Medicine (Baltimore) 102:e33286. 10.1097/md.0000000000033286 36961159 10.1097/MD.0000000000033286 PMC10036044

[22] Mukherjee D, Antar V, Soylemez B et al (2020) High-resolution diffusion tensor magnetic resonance imaging of the brainstem safe entry zones. Neurosurg Rev 43:153–167. 10.1007/s10143-018-1023-4 30136133 10.1007/s10143-018-1023-4

[23] Cohen-Gadol A (2021) Telovelar approach: a practical guide for its expansion to the fourth ventricle. World Neurosurg 148:239–250. 10.1016/j.wneu.2020.12.149 33770846 10.1016/j.wneu.2020.12.149

[24] Lunardi P, Missori P, Gagliardi FM, Fortuna A (1990) Epidermoid tumors of the 4th ventricle: report of seven cases. Neurosurgery 27:532–534. 10.1097/00006123-199010000-00004 2234353 10.1097/00006123-199010000-00004

[25] Yasargil MG, Abdulrauf SI (2008) Surgery of intraventricular tumors. Neurosurgery 62:1029–1040. 10.1227/01.neu.0000333768.12951.9a 18695523 10.1227/01.neu.0000333768.12951.9a

